# Preliminary study: Descriptive analysis of longitudinal data on the international session of the Japan Primary Care Association annual conference, 2012–2018

**DOI:** 10.1002/jgf2.271

**Published:** 2019-08-13

**Authors:** Nobutaka Hirooka, Nobutaro Ban, the Japan Primary Care Association International Affairs Committee

**Affiliations:** ^1^ Department of General Internal Medicine Saitama Medical University Saitama Japan; ^2^ Medical Education Center Aichi Medical University Aichi Japan

**Keywords:** International Session, Japan Primary Care Association, longitudinal analysis

## Abstract

**Introduction:**

Japan Primary Care Association has launched an international session at annual conference since 2012. Longitudinal characteristics of the presentations were investigated.

**Methods:**

Abstracts of the session were analyzed regarding type of presentation (oral/poster), category (research/clinical case/activity/review), and presenters' geographical location.

**Results:**

The numbers of oral and poster presentations significantly increased. The most frequent presentation was research with cross‐sectional survey. Majority of presentations were clinical and less educational research, most of which were by Japanese.

**Conclusion:**

The numbers of presentations in the international session increased, but the session should be used more rigorously to enhance primary care.

## 1 INTRODUCTION

The Japan Primary Care Association (JPCA) was established in 2010 to provide accessible, continual, and comprehensive health care and relevant scientific activities to promote healthy lifestyles.^1^. To accomplish its goals, numerous committee activities are underway,^2^, one of which is the Committee for International Affairs (CIA).

The CIA introduced the international session at its 2012 annual conference. The CIA considered active international interaction critical to the responsible mission of the JPCA and determined that presentation of clinical and research work in English would provide opportunities for researchers to enhance their skills in domestic and international healthcare environments. Session organizers and chairs use the CIA sessions as opportunities for meaningful interactions among primary care physicians and trainees in Japan and other countries.

Trends in the presentations and the characteristics of the participating presenters are important factors to the CIA in its yearly preparation for the annual conference. Thus, the history of the international sessions provides useful insights for the CIA. The CIA encourages active participation in the sessions by domestic and international primary care scholars, and it believes that it is important to share the session goals and the accomplishments derived from the international session to support the participating scholars. This activity is believed to help them use the international session as a future resource.

## 2 METHODS

The data used for the analysis were derived from the abstracts of the presentations given at the international session of the JPCA annual conference from 2012 through 2018. The conference organizer provided the abstracts. Every abstract was reviewed by the primary investigator (N.H.), and the outcome of the review was confirmed by another investigator (N.B.). Following characteristics were identified: (a) type of presentation (oral or poster), (b) category of presentation (research, clinical case, activity, or article review), and (c) presenters' geographic area. The research design was also categorized. A descriptive analysis on the type of presentation, category of presentation, and geographic area was performed. Using regression analysis, the study assessed trend on: (a) the total number of presentations across time, (b) oral or poster type, and (c) presenters' national affiliation.

## 3 RESULTS

There were eight international sessions at the JPCA annual conference. Initially, they were limited to oral presentations and the poster presentation format was not added until 2015. Altogether, 105 presentations were given: 57 were oral (Table S1) and 48 were posters (Table S2). The number of presentations increased over the study period year (Figure S1: *β*_1_ = 4.71, *p* < .05, *R* ^2^ = .71). There is also an increase in the number of oral presentations by year (Figure S2: *β*_1_ = 1.64, *p* < .05, *R* ^2^ **= .66).

### 3.1 Type and category

**Figure 1:**
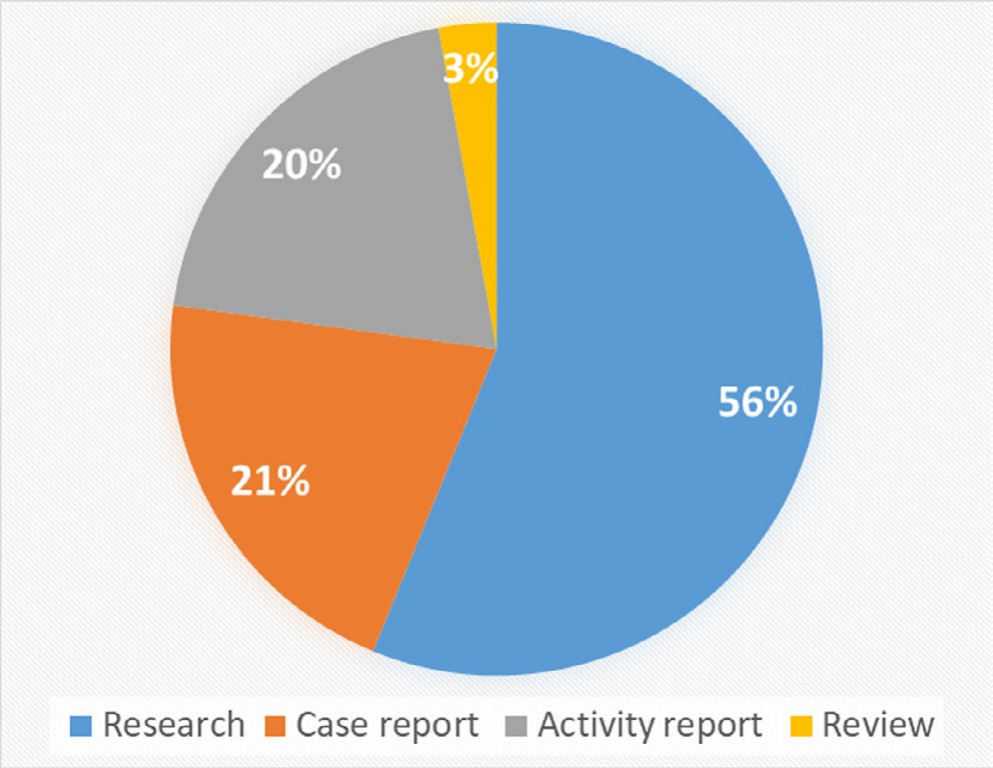
Percentage of presentation by category

Research was the most frequently presented in the session, followed by case report, activity report, and review (Figure 1). Category of presentation in oral and poster was shown as supplements (Tables S1 and S2).

Majority of the research were clinical (71%), and the remainder was educational (21%), and the rest included cost‐effective analysis, evaluation of professional behaviors and evaluation of practice. The most frequent type of research design was cross‐sectional (69%), followed by experimental and cohort design (10% and 5%, respectively). Majority of the educational research employed questionnaire surveys of medical trainees or participants (75%). Regarding the analysis, 55% was quantitative, 29% was qualitative, and the remaining 16% included mixed methods and those with no analysis (methodology presentation).

### 3.2 Geographic area of practice or research

Table 1 shows the numbers of presentations by country. The numbers of presenters from Japan and those from other countries each significantly increased every year. The results of the regression analysis indicate that the increases were by 3.67 (Japan) and 1.03 (other countries) each year.

**Table 1:** Number of presentations by country

Country	N
Japan	82
Singapore	5
U.S.A.	5
Indonesia	4
South Korea	2
Taiwan	2
Iran	1
New Zealand	1
Saudi Arabia	1
Thailand	1
U.K.	1

Abbreviations: U.K., United Kingdom; U.S.A., United States of America.

## 4 DISCUSSION

This study found that the numbers of presentations at the international sessions increased every year (Figure S1 and Table 1), suggesting a global need to enhance and interest in primary care research and development. The purpose of the international session is to enhance active global collaboration. It is important to gain academic support and exchange of expertise in primary health care that is to improve public health and health care.^3^, ^4^ Our efforts to promote international collaborations through the international sessions accelerate this global goal attainment.

Based on the results, it is timely to consider higher quality research activities for presentation in the international session. Methodological robustness is important to research quality, but the designs used for primary care research might not conform to the established approaches, such as randomized controlled trials and prospective cohort studies. Our results found that more than two‐thirds (69%) of the research used cross‐sectional questionnaire. These designs clearly can be improved to produce higher quality evidence. Further, more methodological variety is needed to answer many of the clinical questions in the primary care field because of the breadth of primary health care. A previous study found that an international research network contributed to research capability.^5^ The JPCA's annual conference international sessions should support this network building.

Although the numbers of presenters from around the world with Japanese majority increased, Japanese presentations increased more than foreign ones (Table 1). We should appeal to our international colleagues, particularly to those in the Asia‐Pacific regions to promote international interactions in Asia, where scholarly activity has been less productive than in North America or Europe.^6^ This might boost collaboration and motivation for progress in the scientific aspect of primary care in the target region.

## 5 CONCLUSIONS

We are encouraged by the significant annual increases of international presentations in the international session of the JPCA annual conferences, but several concerns emerged from this study that need to be solved. The international session should be used more for scholarly networking, as methodological learning opportunities, and to present educational research. These weaknesses might be addressed by enriching future sessions, which also might enhance primary care.

## CONFLICT OF INTEREST

The authors have stated explicitly that there are no conflicts of interest in connection with this article.
