# Supplementary figures and images for: Preliminary study: Descriptive analysis of longitudinal data on the international session of the Japan Primary Care Association annual conference, 2012–2018
Source: J Gen Fam Med. 2019 Aug 13;20(6):257–9. doi: 10.1002/jgf2.271 (PMC6875655; doi:10.1002/jgf2.271)

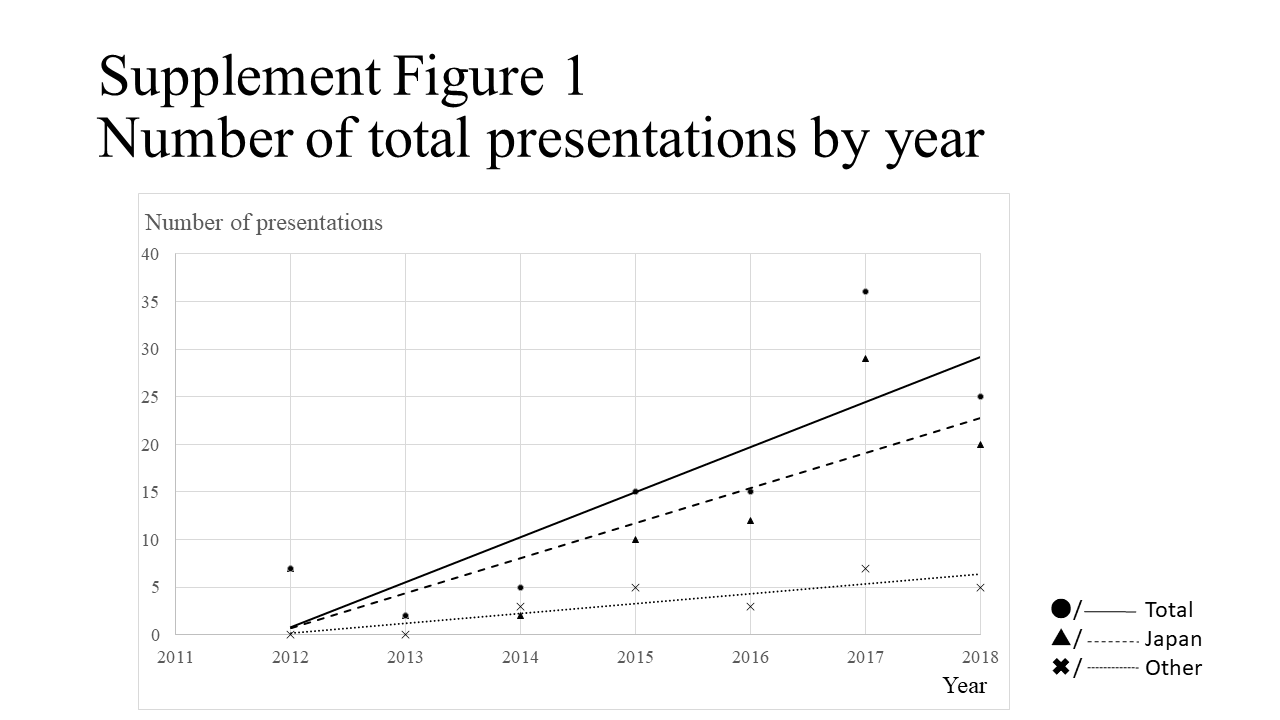

Supplement: Supplementary file 1 [file JGF2-20-257-s001.TIF]

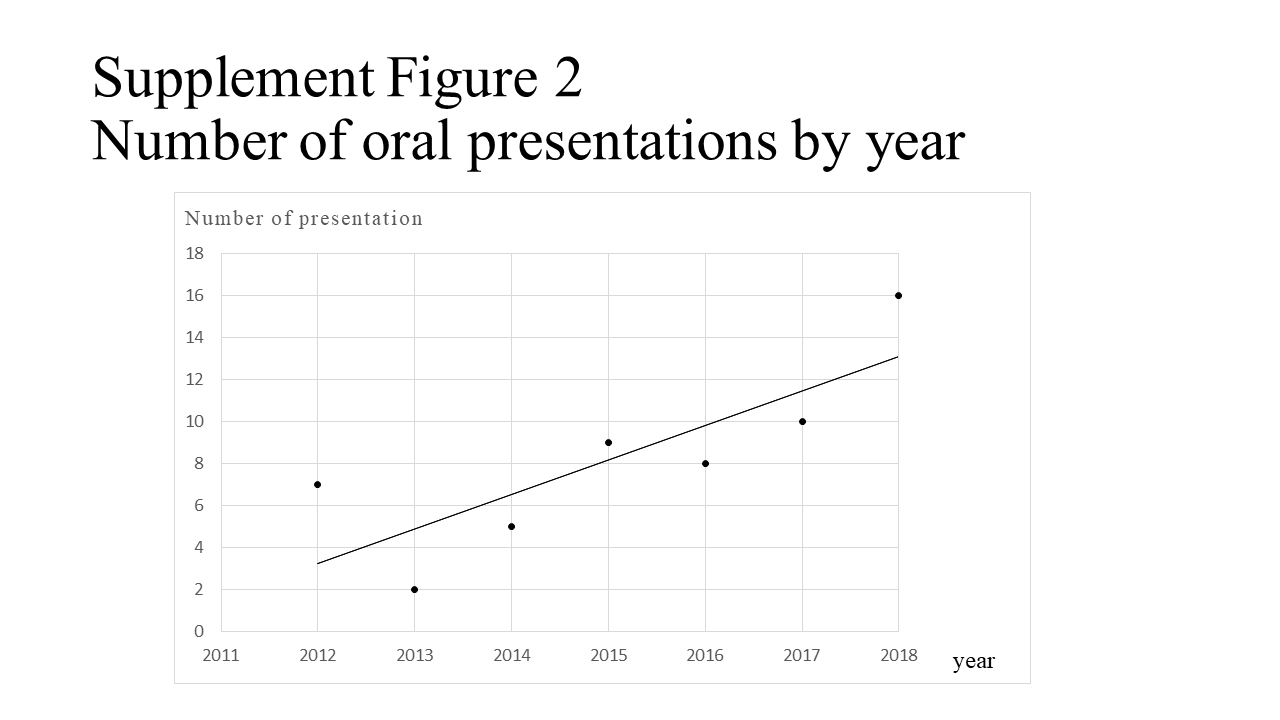

Supplement: Supplementary file 2 [file JGF2-20-257-s002.TIF]
